# Metronomic Chemotherapy for Children in Low- and Middle-Income Countries: Survey of Current Practices and Opinions of Pediatric Oncologists

**DOI:** 10.1200/JGO.18.00244

**Published:** 2019-07-01

**Authors:** Gabriel Revon-Rivière, Shripad Banavali, Laila Heississen, Wendy Gomez Garcia, Babak Abdolkarimi, Manickavallie Vaithilingum, Chi-Kong Li, Ping Chung Leung, Prabhat Malik, Eddy Pasquier, Sidnei Epelman, Guillermo Chantada, Nicolas André

**Affiliations:** ^1^ Assistance Publique–Hôpitaux de Marseille, La Timone Hospital, Marseille, France; ^2^ Metronomics Global Health Initiative, Marseille, France; ^3^ Tata Memorial Hospital, Mumbai, India; ^4^ Homi Bhabha National Institute, Mumbai, India; ^5^ Rabat Children Hospital, University Mohamed V, Rabat, Morocco; ^6^ Dr Robert Reid Cabral Children’s Hospital, Santo Domingo, Dominican Republic; ^7^ Lorestan University of Medical Science, Khorramabad, Iran; ^8^ Netcare Parklands Hospital, Durban, South Africa; ^9^ Prince of Wales Hospital, Chines University of Hong Kong, Sha Tin, People’s Republic of China; ^10^ The Chinese University of Hong Kong, Sha Tin, People’s Republic of China; ^11^ All India Institute of Medical Sciences, New Delhi, India; ^12^ Centre de Recherche en Cancérologie de Marseille, Centre National de la Recherche Scientifique, Institut National de la Santé et de la Recherche Médicale, Aix-Marseille Université, Institut Paoli-Calmettes, Marseille, France; ^13^ Santa Marcelina Hospital, São Paulo, Brazil; ^14^ Hospital JP Garrahan, Buenos Aires, Argentina

## Abstract

**PURPOSE:**

Low- and middle-income countries (LMICs) experience the burden of 80% of new childhood cancer cases worldwide, with cure rates as low as 10% in some countries. Metronomics combines frequent administrations of low-dose chemotherapy with drug repurposing, which consists of using already-approved drugs for new medical applications. With wide availability, limited costs, and little infrastructure needs, metronomics can be part of constraint-adapted regimens in these resource-limited settings—with the understanding that metronomics shall not be a substitute for standard treatments when available and doable. Our study aims to describe the experience, practices, opinions, and needs in metronomics of physicians working in LMICs.

**METHODS:**

An online questionnaire was sent to more than 1,200 physicians in pediatric oncology networks in LMICs. Items included the type of center, physician’s demographics, experience in pediatric oncology, and experience with current knowledge of metronomics. Opinions and perspectives were explored using multiple-answer and open questions.

**RESULTS:**

Of physicians, 17% responded. Of respondents, 54.9% declared that they had already used a metronomic regimen. The most frequently cited repositioned drugs were celecoxib (44%) followed by propranolol and valproic acid (17%). Respondents highlighted the advantages of outpatient use (20%) and expected low toxicity (24%). In considering the drawbacks of metronomics, 47% of responses highlighted the lack of scientific evidence or guidelines, 33% the availability or affordability of drugs, and 18% the problem of acceptance or compliance. Of physicians, 79% believed that use of metronomics will spread in LMICs in the near future and 98% of them were willing to participate in international metronomic protocols or registries.

**CONCLUSION:**

Metronomics is already used in LMICs and is a potential answer to unmet needs in pediatric oncology. There is room for improvement in the availability of drugs and a necessity to develop collaborative protocols and research to generate level A evidence.

## INTRODUCTION

Despite major improvements in treatment and cure rates, childhood cancer remains a challenge for physicians worldwide.^1,2^ In 2015, low- and middle-income countries (LMICs) represented 139 economic areas, more than 6 billion people,^3^ and 80% of new childhood cancer cases worldwide, with cure rates as low as 10% in some countries.^4,5^ Thus, improvement of childhood cancer treatment in LMIC seems to be the most important challenge in terms of survival and quality of life.

In high-income countries, a cure rate of 80% is achieved through early diagnosis and prognosis stratification, along with timely multimodality treatment that consists of surgery, systemic therapy (chemotherapy and targeted therapies), and radiation therapy where indicated. These resources are sparse and unequally available in LMICs. In addition, because of overcrowding and a lack of antibiotic stewardship in LMICs, there is a higher incidence of multidrug-resistant infection that requires the use of more antibiotics and increases the costs of supportive care.^6^

Improving medical care for children with cancer in LMICs requires political will. Pediatric cancer plans should be created with a focus on development of registries, improvement of medical education and international cooperation, and development of tertiary care centers dedicated to childhood cancer.^7^ Meanwhile, physicians in LMICs need more affordable and constraint-adapted medical options.^8^ Aside from maximum tolerated dose (MTD) chemotherapy as practiced in high-income countries, adapted regimens have been developed to allow for treatments with a favorable risk–benefit balance in resource-limited settings.^9,10^ Absolute priority should be given to achieving availability of standard chemotherapy drugs that have proven efficacy in low-resource settings following adapted protocols.

**CONTEXT**

**Key Objective**
To describe experience, practices, opinions, and needs with regard to metronomics of physicians working in pediatric oncology in low- and middle-income countries (LMICs) where specific constraints can limit the availability and feasibility of standard treatments.
**Knowledge Generated**
Both metronomic chemotherapy and drug repositioning are used by pediatric oncologists working in LMICs who have a good knowledge and understanding of associated mechanisms of action and potential advantages or drawbacks.
**Relevance**
Metronomics is already being used in LMICs and is a potential answer to unmet needs in pediatric oncology. There is room for improvement in the availability of drugs as well as a necessity to develop collaborative protocols and research to generate level A evidence.

Metronomics combines metronomic chemotherapy (MC) with drug repurposing.^8^ MC relies on the administration of chemotherapy on a frequent schedule at doses that are less than the MTD, with nonprolonged drug-free breaks. Drug repurposing consists of using already-approved drugs for which anticancer properties have been unveiled, leading to new medical applications. These treatments are widely available—and can be on the WHO essential drug list—have limited costs, need little care infrastructure, and may also have lower dropout rates.^8^ Metronomics could be considered as a constraint-adapted alternative treatment when evidence for the standard adapted regimen is lacking and/or not feasible.

The effectiveness of MC has been investigated in adults in large, phase III clinical trials with promising results.^11-13^ In children, most studies are phase I and II trials for refractory disease^14-18^ or, less frequently, as neoadjuvant therapy.^19,20^ Recently, phase III studies of metronomics as palliative treatment in LMICs have been published^21,22^ which have confirmed a growing interest in this therapeutic option.

The metronomics approach may fit LMIC constraints in different settings. This approach may work as maintenance treatment in patients with high-risk neoplasms—when intensification or local treatment is not available—or more rarely as a bridge to additional treatment—that is, hematopoietic stem-cell transplantation or immunotherapy—if available in a tertiary twinned center. This approach may also be a part of first-line therapy for patients with poor general condition or when poor compliance to treatment is anticipated, or as palliative treatment of high-risk, refractory, and relapsing diseases.

Considering the incidence of pediatric cancer and the lack of resources in some countries, it seems that evidence of feasibility, efficacy, and tolerance of metronomics would mostly come from LMICs. In this context, our study aims to describe the experience, practices, opinions, and needs of physicians working in LMICs with regard to metronomics.

## METHODS

This study was conducted from May 2016 to December 2016 using an online questionnaire that was sent to more than 1,200 physicians. Practitioners were invited to participate by the intermediate of a national reference from the Metronomic Global Health Initiative or pediatric oncology networks, such as Cure4Kids, Asociación de Hemato-Oncología Pediátrica de Centro America, Société Marocaine d’Hématologie et d’Oncologie Pédiatrique, Groupe Franco-Africain d’Oncologie Pédiatrique, China Children Cancer Group, South African Children's Cancer Study Group, and the Pediatric Hematology Oncology Chapter of Indian Academy of Pediatrics.

The questionnaire was generated using the Qualtrics platform and was anonymous. It contained multiple answer questions (MAQs) and open questions. Items included the type of center and physician demographics and experience in pediatric oncology. Current knowledge of metronomics was assessed by asking participants to rank the mechanisms of action from important (1) to secondary (3) and by an open question about repositioned drugs—that is, “Quote up to four repositioned drugs”.

Experience was evaluated by the number of patients who were treated with metronomics per year and MAQs about pathologies, clinical situations, and protocols that apply to the use of metronomics. Clinical situations in which metronomics could be indicated had to be rated from likely (1) to not likely (3).

Opinions and perspectives were explored by MAQs and open questions that evaluated the physician’s judgment about the main advantages, drawbacks, and local obstacles of the use of metronomics. Finally, we asked binary questions about the future development of metronomics and willingness to develop its prescription and to participate in international protocols or registries.

### Statistical Analysis

Results were analyzed as a single sample and no comparisons between countries, demographics, experience, or type of center were conducted. Results were presented as the percentage of items over the total of responses in MAQs, as the mean score or rank for each variable, and as the number of occurrences of drug citation or concept for open questions.

## RESULTS

**FIG 1:**
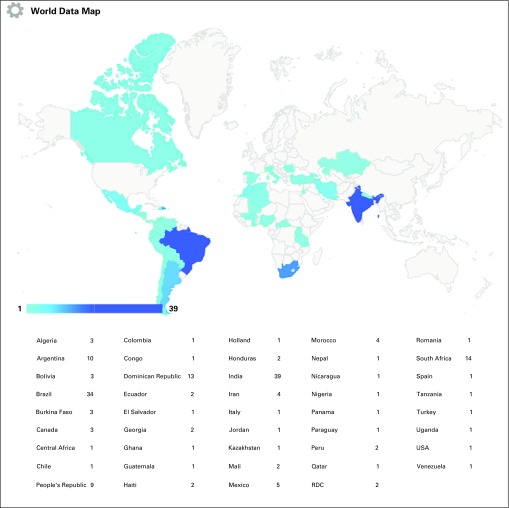
Number of respondents by country. CAR, Central African Republic; DRC, Democratic Republic of Congo.

Of respondents, 211 submitted a complete or incomplete survey. The mean questionnaire response rate was 17%. Characteristics of the respondents are detailed in Table 1. Most responses came from South America (29.1%; 18.6% from Brazil alone), India (21.4%), Central America (12.5%), and sub-Saharan Africa (11.4%). Geographic distribution is detailed in Figure 1.

**TABLE 1:**
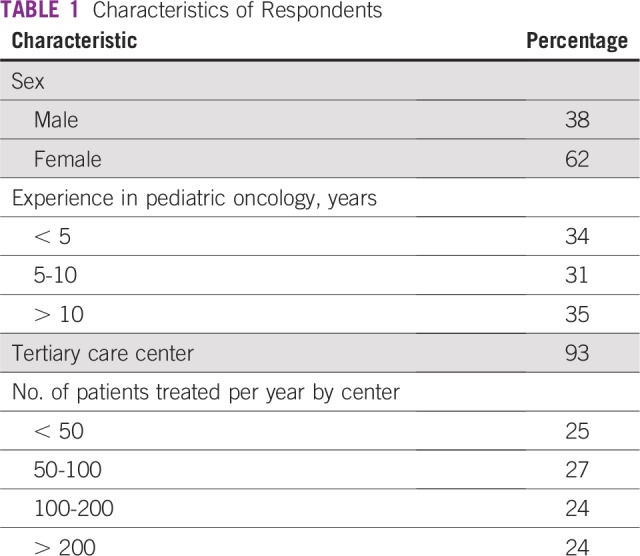
Characteristics of Respondents

### Current Knowledge About Metronomics

Of respondents, 39.3% had first heard about metronomics in scientific and medical meetings, 30.9% from colleagues, and, to a lesser extent, through publications (11%) or clinical trials (11%). Mechanisms of action of MC were rated with a minimum score of 1 for (important) and a maximum score of 3 (secondary). Mean scores were 1.67 for antiangiogenic effect, 1.90 for activation of antitumor immunity, 2.07 for effect on cancer stem-like cells, and 2.14 for direct toxicity on cancer cells.

**FIG 2:**
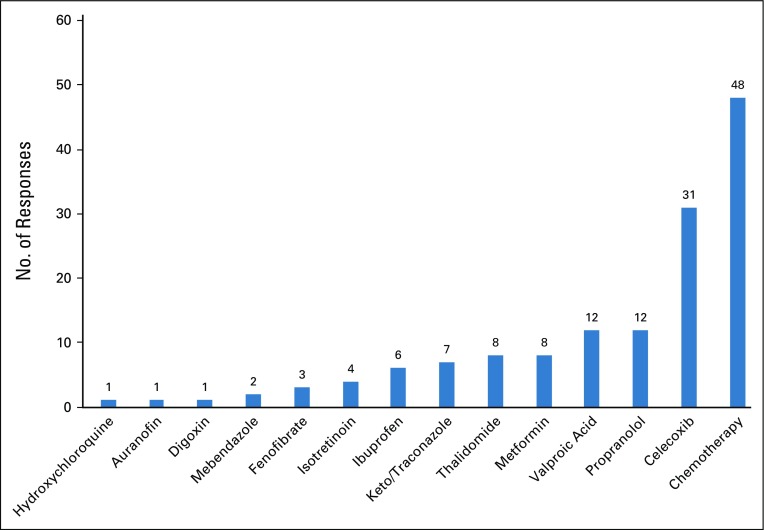
Occurrence of repositioned drugs and chemotherapy citation.

More than 50% of respondents declared that they were familiar with drug repurposing. When asked to quote up to four repositioned drugs, 56% of the 73 respondents cited a repositioned drug, whereas others cited cytotoxic drugs used in standard MC regimens. Citations of repositioned drugs or chemotherapy are shown in Figure 2. Celecoxib was the most frequently cited, with close to 44% of participants quoting, followed by propranolol and valproic acid (17%) and metformin, thalidomide, keto/itraconazole, and ibuprofen (approximately 10%).

### Experience in Prescribing Metronomics

**FIG 3:**
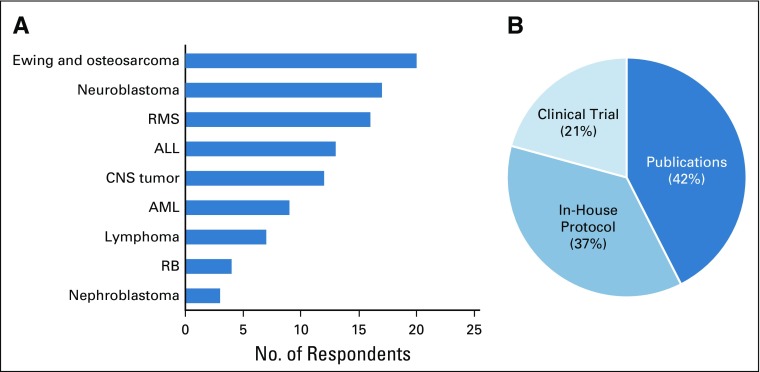
Current use of metronomics. (A) Indication, percent of responses (n = 116 respondents). (B) Type of protocol, percentage of responses (n = 106 respondents). AML, acute myeloid leukemia; RB, retinoblastoma; RMS, rhabdomyosarcoma.

Of 211 physicians, 116 (54.9%) declared that they had used a metronomic regimen at least once. Of those who had used metronomic therapy, 67.5% treated fewer than 10 patients per year with metronomics. One fifth of respondents (21%) reported treating more than 20 patients a year with this approach. The majority of clinicians (73.3%) used a preexisting metronomic protocol rather than a personalized one. Diseases for which a metronomic therapy was initiated and the frequency of different types of protocols are described in Figures 3A and 3B, respectively.

Indications for metronomics are presented by mean scores (from a minimum score of 1 [very likely] to 3 [not likely]): 2.54 for first line treatment, 1.86 for maintenance therapy, 1.53 for relapse or refractory disease, and 1.35 for palliative purposes.

### Physicians’ Opinions on Metronomics

**FIG 4:**
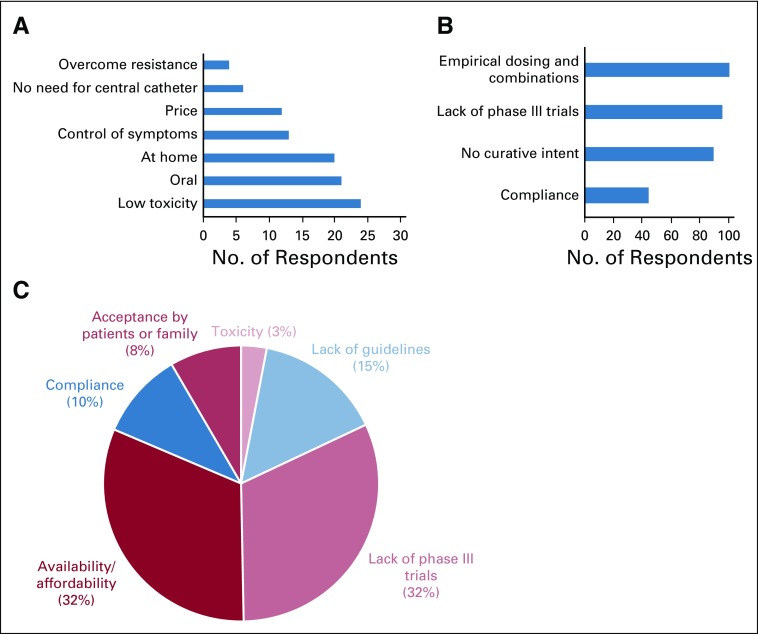
Advantages and drawbacks of metronomics and obstacles to its use. (A) Main advantages of metronomics, percentage of responses (n = 149 respondents). (B) Main drawbacks, number of responses (n = 93 respondents). (C) Obstacles to using Metronomics in respondents’ settings, percentage of citations (n = 107 citations).

Different opinions about the advantages and drawbacks of metronomics are presented in Figure 4. The most frequent statements on the advantages of metronomics were the possibility of being used at home and the expected low toxicity. Control of symptoms and the possibility of overcoming of drug resistance were less frequently cited.

Responses to the open question about obstacles to the use of metronomics by physicians in their own settings have been summarized in Figure 4. Responses highlighted the lack of scientific evidence or guidelines (47% of responses) to support and guide the use of metronomics. Of interest, the availability or affordability of drugs was a frequent concern (almost 33% of responses) and 18% of responses mentioned the problem of acceptance or compliance.

### Perspectives

A majority of respondents (79%) reported that they believe that the frequencies of metronomics use would increase in LMICs and in their practices in the future. In addition, almost all respondents (98%) were willing to participate in international metronomic protocols or registries.

## DISCUSSION

Our survey addresses the relevance of metronomics in pediatric oncology as a potential constraint-adapted therapy for pediatric cancer in LMICs. Data show that pediatric oncologists in LMICs are familiar with the concepts of both MC and drug repurposing, although specific knowledge about drug combinations and indications must be improved. Lack of preclinical evidence for dosing and combinations as well as an insufficient number of phase III trials were the most important concerns to physicians.

MC is widely used as maintenance therapy after achieving complete remission in leukemia for which it has been demonstrated to put the endothelium to rest in children with acute lymphoblastic leukemia.^23^

In patients with high-risk solid tumors, several studies have documented a potential interest. In high-risk neuroblastoma, the German Society for Pediatric Oncology and Hematology reported in a randomized controlled trial that megatherapy or MC maintenance demonstrated similar overall survival at 3 years, whereas megatherapy led to better event-free survival.^24^ Therefore, in LMIC centers in which high-dose chemotherapy is not available, MC maintenance could represent a genuine alternative. In patients with metastatic Ewing sarcoma, adding metronomics maintenance with daily celecoxib, weekly vinblastine, and daily oral cyclophosphamide after standard chemotherapy led to better 3-year overall survival (0.6 *v* 0.2) and 3-year event-free survival (0.5 *v* 0.2).^25^ More recently, Bisogno et al^26^ reported improved overall survival with 87% (*v* 77%; *P* = .011) in a randomized controlled trial of maintenance MC of six 28-day cycles of intravenous vinorelbine and continuous daily oral cyclophosphamide among 371 patients with high-risk nonmetastatic rhabdomyosarcoma. Last, in patients with desmoplastic small round-cell tumors, MC was associated with more frequent complete remission and longer event-free survival.^27^

Metronomics as an upfront therapy is likely limited by the lack of supporting evidence. Data are scarce, but there are studies that support this approach in specific indications. In India, Lakshmaiah et al^20^ reported 66.7% event-free survival with an upfront vinblastine metronomic regimen for anaplastic large-cell lymphoma. In Canada, Lassaletta et al^28^ reported 53.2% progression-free survival in children with low-grade glioma that was treated with weekly vinblastine. This result is similar to that obtained with MTD carboplatin-vincristine standard chemotherapy with improved toxicity profile. These results suggest that metronomic regimens can benefit patients receiving first-line treatment regardless of the economic context. In addition, the recent availability of oral vinca-alkaloids may extend the scope of indications.

Considering palliative indications, bone tumors were cited as the most frequent indication for metronomics. This may be explained by frequent metastatic presentation at diagnosis, nonoptimal access to extensive surgery, and a lack of sufficient supportive care to allow for MTD chemotherapy regimens that contain high-dose methotrexate, cisplatin, or doxorubicin. However, recent evidence suggests that the efficacy of metronomics in osteosarcoma is limited compared with other neoplasms.^21,22^ Thus, Pramanik et al,^21^ in a recent randomized controlled trial, intended to demonstrate that a four-drug oral regimen—that is, daily celecoxib and thalidomide and alternating oral etoposide and oral cyclophosphamide—was beneficial in extra-CNS pediatric solid tumors. Although global progression-free survival did not seem to be modified compared with best supportive care, subpopulation analysis that excluded bone sarcoma demonstrated improved progression-free survival for the group that received MC.^21^ Unawareness of this emerging limit to metronomic regimens may lead to the perception of a lack of efficacy and therefore weaken the efforts at implementation of metronomic protocols with a palliative purpose.

Metronomics seems to be a potential answer to some unmet need in pediatric oncology^29^ and seems to be chosen mostly for palliative purposes or uncontrolled situations. Furthermore, advantages of metronomics endorsed by pediatric oncologists were mostly low toxicity (24%), at-home treatment (20%), and oral administration (21%) compared with other aspects, like possibly overcoming drug resistance (4%) or better control of symptoms (13%). These data suggest that the main expectations for metronomics focus on low constraints for patients rather than the expected efficiency of symptom or disease control.

Despite potential interest in metronomics, empirical dosing and combination were identified as major drawbacks by all respondents to MAQs. Likewise, 47% of responses to the open question identified the lack of phase III trials or guidelines as obstacles to prescription.

Preclinical and clinical research must be pursued to build a stronger rationale for combinatorial treatment. Such work has recently been described in new therapeutic research for angiosarcoma with compelling results.^30^

Despite its simple design, our study is exposed to selection and participation bias by different means. Physicians who were contacted by local referents and who completed the survey were likely already familiar with metronomics and trained by these metronomics network referents. Second, our physician sample is influenced by the diverse origins of participants. Physicians in LMICs experience different economic and sanitary constraints between countries and between different settings in the same country—as for example, from rural to urban areas or in tertiary care centers compared with others. Last, some physicians were contacted by their origin country’s network but work in high-income settings. These respondents might have reported opinions and practices that may not fit with constraints in LMICs.

Our study shows that general knowledge about metronomics is accurate and indicates that there is a willingness of pediatric oncologists to develop this strategy in LMICs. It seems that there is room for improvement in the availability of drugs and a necessity to develop collaborative protocols and research to generate level A evidence. There is an opportunity for dramatic improvement of the health status of numerous children and adolescents with cancer in LMICs through a constraint-adapted approach. Developing metronomic registries and protocols is a mandatory intermediate step.
